# P-1653. Altered Gut Microbiome Composition in Early Childhood COVID-19

**DOI:** 10.1093/ofid/ofaf695.1828

**Published:** 2026-01-11

**Authors:** Byung Ok Kwak, Dong Hyun Kim, Ky Young Cho

**Affiliations:** Inha University School of Medicine, Incheon, Inch'on-jikhalsi, Republic of Korea; Inha University School of Medicine, Incheon, Inch'on-jikhalsi, Republic of Korea; Hallym University Kangnam Sacred Heart Hospital, Seoul, Seoul-t'ukpyolsi, Republic of Korea

## Abstract

**Background:**

The gut microbiome plays a crucial role in immune function, and its dysregulation may influence the clinical course of infectious diseases, including coronavirus disease 2019 (COVID-19). Early childhood is a critical period for gut microbiome development, yet data on gut microbiome alterations in young children with COVID-19 remain limited. This study aimed to characterize gut microbiome changes in young children infected with severe acute respiratory syndrome coronavirus 2 (SARS-CoV-2).

**Methods:**

Stool samples were collected from 18 children under 2 years of age with confirmed SARS-CoV-2 infection and 7 healthy controls between December 2021 and June 2022. Gut microbiota composition was analyzed using 16S rRNA gene sequencing.

**Results:**

In children with COVID-19, the gut microbiome exhibited an increase in Bacteroidetes and Firmicutes, whereas Actinobacteria and Proteobacteria were reduced, with higher abundances of *Bifidobacterium*, *Escherichia*, and *Streptococcus* and lower abundances of *Faecalibacterium*, *Clostridium*, and *Ruminococcus* compared to healthy controls. Children with COVID-19 exhibited reduced alpha diversity, indicating microbial dysbiosis, and significant differences in beta diversity compared to healthy controls. Several immune-related pathways, including NOD-like receptor, IL-17, Fc gamma R-mediated phagocytosis, Th17 differentiation, and Toll/Imd signaling, were significantly downregulated in the gut microbiome of children with COVID-19.

**Conclusion:**

This study highlights significant alterations in the gut microbiome of young children with SARS-CoV-2 infection, emphasizing the need for further research into the potential long-term effects of gut dysbiosis on child health and development.

**Disclosures:**

All Authors: No reported disclosures

